# Cytokine storm-calming property of the isoquinoline alkaloids in *Coptis chinensis* Franch

**DOI:** 10.3389/fphar.2022.973587

**Published:** 2022-09-06

**Authors:** Yuejia Lan, Huan Wang, Jiasi Wu, Xianli Meng

**Affiliations:** ^1^ State Key Laboratory of Southwestern Chinese Medicine Resources, Innovative Institute of Chinese Medicine and Pharmacy, Chengdu University of Traditional Chinese Medicine, Chengdu, China; ^2^ Acupuncture and Tuina School, Chengdu University of Traditional Chinese Medicine, Chengdu, China

**Keywords:** cytokine storm, inflammatory signaling pathway, *Coptis chinensis* franch, isoquinoline alkaloid, berberine

## Abstract

Coronavirus disease (COVID-19) has spread worldwide and its effects have been more devastating than any other infectious disease. Importantly, patients with severe COVID-19 show conspicuous increases in cytokines, including interleukin (IL)-6, monocyte chemoattractant protein (MCP)-1, IL-8, tumor necrosis factor (TNF)-α, IL-1, IL-18, and IL-17, with characteristics of the cytokine storm (CS). Although recently studied cytokine inhibitors are considered as potent and targeted approaches, once an immunological complication like CS happens, anti-viral or anti-inflammation based monotherapy alone is not enough. Interestingly, certain isoquinoline alkaloids in *Coptis chinensis* Franch. (CCFIAs) exerted a multitude of biological activities such as anti-inflammatory, antioxidant, antibacterial, and immunomodulatory etc, revealing a great potential for calming CS. Therefore, in this timeline review, we report and compare the effects of CCFIAs to attenuate the exacerbation of inflammatory responses by modulating signaling pathways like NF-ĸB, mitogen-activated protein kinase, JAK/STAT, and NLRP3. In addition, we also discuss the role of berberine (BBR) in two different triggers of CS, namely sepsis and viral infections, as well as its clinical applications. These evidence provide a rationale for considering CCFIAs as therapeutic agents against inflammatory CS and this suggestion requires further validation with clinical studies.

## 1 Introduction

In 2019, the first outbreak of coronavirus disease (COVID-19) led to a serious public health event which threatened global health. According to a *Lancet* report, the main cause of COVID-19 death is acute respiratory distress syndrome (ARDS) (Hu et al., 2021). Moreover, a growing body of clinical data suggests that cytokine storm (CS) and inflammatory signaling pathway transduction are two crucial factors contributing to ARDS in patients with COVID-19 (Choudhary et al., 2021). CS not only worsens the severity of infection, but also affects the heart, liver, kidney, gastrointestinal system, and the central nervous system, eventually leading to multiorgan failure (MOF) (Ye et al., 2020). Therefore, early recognition and timely treatment of CS is of great significance for treating critical patients and for reducing the mortality rate.

CS is an inflammatory syndrome in which cytokines are abnormally released in response to infection and other stimuli. During the process, a mass of pro-inflammatory cytokines and growth factors, typically including IL-18, IL-6, IL-17, IL-1β/-1α, IFN-γ, and TNF-α, as well as chemokines are released to fuel the CS (Huang et al., 2020), (Zhu et al., 2020). Meanwhile, a population of immune cells is infected during this process, causing a sustained inflammatory response (Channappanavar and Perlman, 2017). Thus, controlling the inflammatory response by immunomodulators and reducing or antagonizing cytokine levels are effective measures to calm CS (Weckmann and Alcocer-Varela, 1996), (Ye et al., 2020). At present, various of cytokine inhibitors have been applied in the CS treatment, including IL-6-antagonists (i.e., siltuximab), IL-1-antagonists (i.e., anakinra), IL-17-antagonists (i.e., secukinumab), TNF-α-blockers (i.e., infliximab), and INF-α-inhibitors (i.e., sifalimumab), as well as immunomodulators glucocorticoids (Li et al., 2020b), (Ye et al., 2020). However, it is worth noting that these drugs are a double-edged sword in the context of antiviral infections. Although the therapeutic effect of inhibitors is remarkable, their application has some unavoidable adverse reactions. For example, tocilizumab posed mild liver test disturbances (Jamilloux et al., 2020). Additionally, prolonged indiscriminate suppression of inflammation raises concerns about the ability to clear the pathogen, as well as the increased risk of secondary infection. For example, the recipients of emapalumab occurred (bacterial, viral, and opportunistic) infections and multiple organ dysfunction syndrome (Jamilloux et al., 2020). Consequently, for treating viral infection and excessive inflammatory complications, drugs that reduce inflammation and modulate innate immune response without compromising the adaptive immune response may more effectively manage CS patients (Manjili et al., 2020).

*Coptis chinensis* Franch. (CCF) (Huang Lian), is a widely used traditional Chinese herbal medicine, which has been reported to exhibit antibacterial, anti-oxidant, anti-hyperglycemic, and anti-inflammatory activities (Meng et al., 2018). We traced the database of literature published within the last decade and found that isoquinoline alkaloids from CCF demonstrate potential to calm CS. For instance, Berberine (BBR), coptisine (COP), and palmatine (PAL) all belong to the CCF isoquinoline alkaloids (CCFIAs) (Meng et al., 2018), as well as the anti-inflammatory compounds of CCF (Li et al., 2015). CCFIAs inhibit the production of the inflammatory cytokines and mediators such as IL-1α/β, IL-6, IL-17, IFN-γ, TNF-α, nitric oxide (NO), prostaglandins (PGs), leukotrienes, and reactive oxygen species (ROS) (Li et al., 2015). The molecular mechanisms underlying the immunomodulatory and anti-inflammatory effects of CCFIAs include downregulation of toll-like receptors (TLRs), and inflammation-associated pathways, representative ones include nuclear factor-κB (NF-κB), mitogen-activated protein kinase (MAPK), Januskinase/Signal transducer and activator of transcription (JAK/STAT) and inflammasome NLRP3 (Wang et al., 2019), (Li et al., 2019), (Sun et al., 2019), (Yao et al., 2019).

In this review, we elaborate the underlying molecular mechanisms of CS progress, then we retrieve articles concerning the application of CCFIAs in the treatment of cytokine storm related inflammatory diseases from PubMed, Web of Science and Geenmedical through electronic and manual retrieval, a total of 520 publications were identified, 75 of which were included in this systematic review. Additionally, we present recent experimental data on the inhibition of pro-inflammatory mediators’ production by CCFIAs, as well as compare the similarities and distinctions in mechanisms/effects among CCFIAs. Furthermore, we summarize the therapeutic role in sepsis and viral infections and clinical application of BBR. Given that CCFIAs have antiviral and antibacterial effects in addition to downregulating cytokine production, they may be more promising therapeutic candidates for preventing infection-associated CS than drugs with only antimicrobial or anti-inflammatory activity.

## 2 Cytokine storm

**FIGURE 1:**
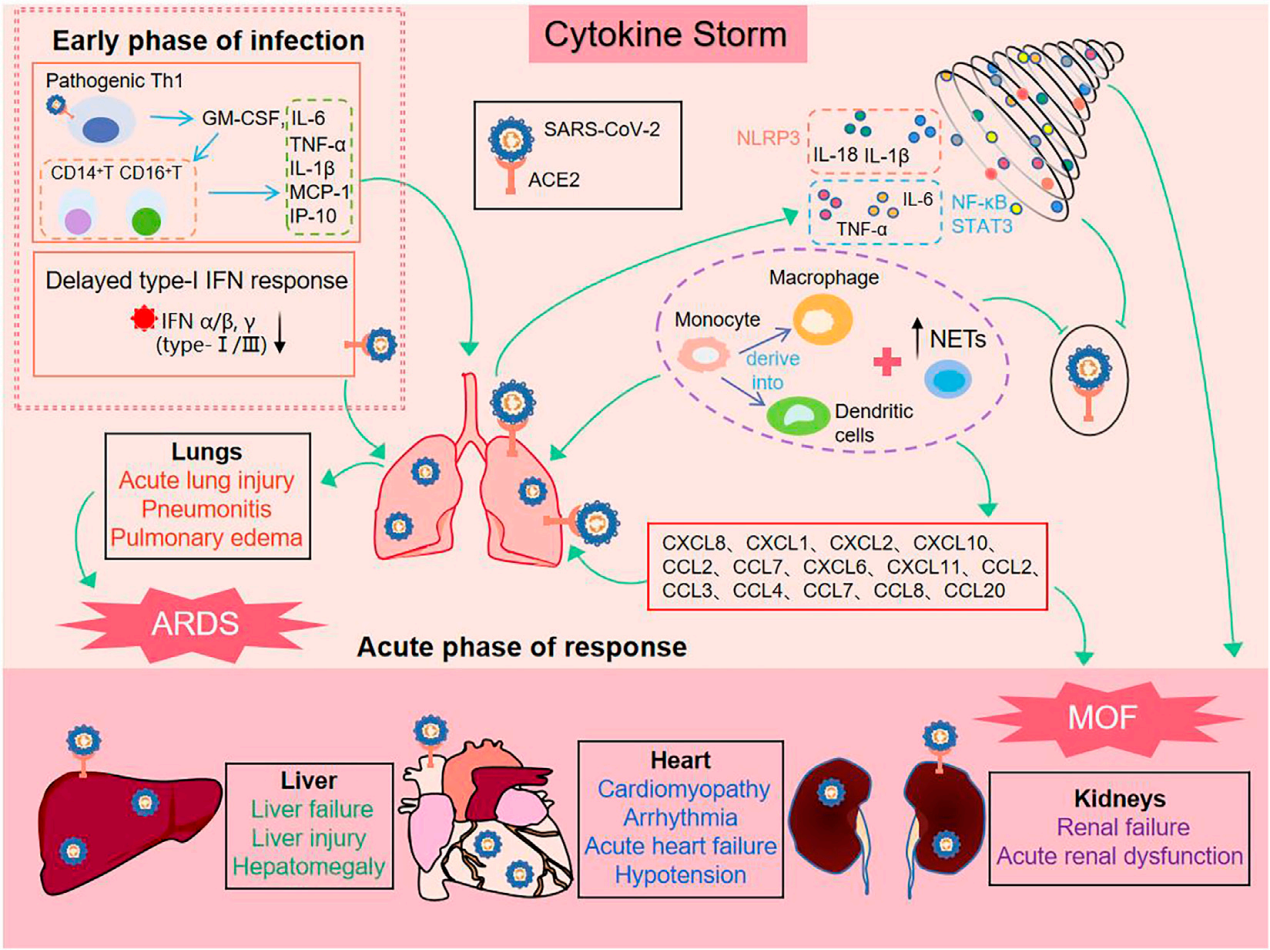
Process of cytokine storm production induced by SARS-CoV-2.

CS is a systemic inflammatory response by a dysregulated immune system which refers to those situations of overly exuberant inflammation leading to critical conditions, such as ARDS, disseminated intravascular coagulation (DIC) or MOF (Jamilloux et al., 2020). In early stages of COVID-19, SARS-CoV-2 enters the host cell and attaches to angiotensin-converting enzyme 2 (ACE2), which is the key participant in the pathogenesis of COVID-19 (Iwasaki et al., 2021) (Shown in Figure 1). Rapidly, activation of the innate immune response and hypercytokinemia occur in COVID-19 patients, activated pathogenic Th1 cells secrete proinflammatory cytokines, such as GM-CSF and IL-6. GM-CSF further activates CD14^+^CD16^+^inflammatory monocytes to produce large quantities of IL-6, TNF-α, and other cytokines (MCP-1, IL-1β, IL-17), followed by recruitment and activation of abundant inflammatory cells, for example, neutrophils form neutrophil extracellular traps (NETs) to facilitate cytokine release with the positive feedback, and monocytes migrate to the lung and further derive into macrophage or monocyte derived dendritic cells (Zhou et al., 2020) (Huang et al., 2020). On the other hand, severe patients have a temporary immunodeficient state *in vivo*, characterized by a delayed type-I IFN response and lymphopenia, which may explain CS and more severe diseases (Huang et al., 2020), (Channappanavar et al., 2016). The delayed secretion of type Ⅰ and Ⅲ IFNs, including IFN α/β leads to an excessive late immune response, and generalized hyper-inflammation in lung that induces acute lung injury (Channappanavar et al., 2016), (Kim et al., 2021). Subsequently, there are lung infiltration by monocytes, macrophages and neutrophils, as well as recruiting mediators. These acute inflammatory mechanisms damage the pulmonary microvascular and alveolar barrier and cause vascular leakage and alveolar edema, converging to ARDS, and initiate CS in the lung (Channappanavar et al., 2016), (Quirch et al., 2020). In all these conditions, IL-1β, IL-18, IFN-γ, and IL-6 are key mediators of hyperinflammation. Finally, chemokines release can attract extra inflammatory cells to migrate into the inflammation site that intensify CS and may have indirect impacts on MOF, specially kidneys, liver, and heart (Ahmadian et al., 2021), (Bavishi et al., 2020), (Alqahtani and Schattenberg, 2020). In addition, ACE2 was expressed in vital organs (lung, heart, intestine, brain, kidney, liver, etc.,). Thus SARS-CoV-2 also directly damages the target organ by binding to ACE2, and then exacerbation by inflammatory responses (Iwasaki et al., 2021).

**FIGURE 2:**
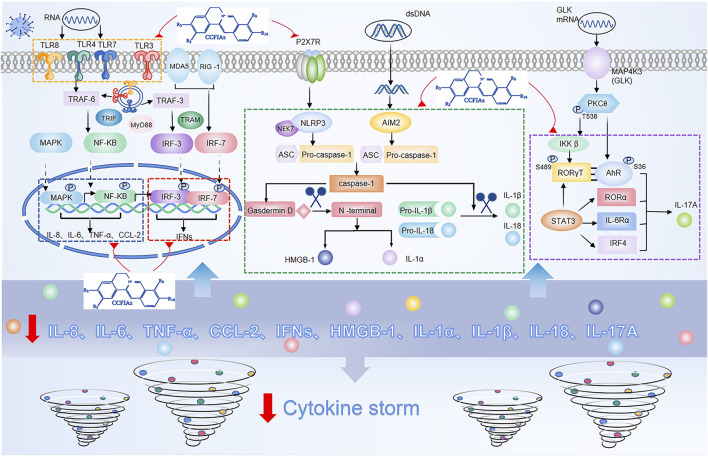
Overactivation of innate immune signaling pathways and modulation of these pathways by isoquinoline alkaloids in *Coptis Chinensis* Franch.

As previously mentioned, cytokines play a central role as inflammatory mediators in CS. Generally, cytokine secretion is mediated by three pathways, namely 1) the angiotensin II/angiotensin receptor type 1 (AT1R) pathway (Ni et al., 2020); 2) the ACE2 signaling pathway (Chen et al., 2010); and 3) innate immune signaling pathways, including pattern recognition receptors (PRRs) such as TLRs, RIG-1 (Park and Iwasaki, 2020), and inflammasomes containing NLRP3 (Shah, 2020), AIM2 (Junqueira et al., 2021). Among these, the activation of the innate immune response system is the most difficult to control and meanwhile the easiest way to potentiate excessive release of cytokines. In principle, controlling an ongoing inflammatory response by specifically or nonspecifically targeting inflammatory cytokines or related signaling pathways can be considered a promising choice for therapeutic strategy for CS. Crucial roles for the inflammatory signaling pathways and downstream cytokines are shown in (Figure 2).

In response to viral infection, PRRs recognize and bind to pathogen-associated molecular patterns (PAMPs), triggering activation of the innate immune response system of the invading virus (Medzhitov, 2007). Among PRRs, the most typical ones are TLRs, whose activation eventually causes IRF3, NF-ĸB, MAPK, and JAK/STAT to be activated (Lim and Staudt, 2013). One of the major pathways for NF-ĸB activation after virus infection is via the MyD88, the other is the angiotensin II(Ang II) pathway. Ang II via AT1R activates NF-κB, and metalloprotease 17 (ADAM17), which generates the mature form of TNF-α (Fara et al., 2020). Subsequently, ADAM17 induces STAT3 activation, which coactivates the IL-6 amplifier (AMP) with NF-κB, and further activates various proinflammatory factors, such as IL-6, IL-8, MCP-1, and VEGF (Cortese et al., 2020). In addition, similar with NF-κB, MAPK also mainly regulates the release of IL-6 and TNF-α (Asiedu et al., 2021). With regard to the route of IFN-γ release, IRF3 is widely expressed. Besides, IRF3 also directly induces the expression of cytokines other than type I IFNs, including CXCL10, IL-12, IL-23, and IL-15 (Brownell et al., 2014), (Koshiba et al., 2013). In addition to lL-6 activation combined with NF-κB, STAT3 also directly activates IL-17A, and acts in conjunction with MAP4K3 to selectively promotes IL-17A transcription by inducing the AHR–ROR–γ T complex (Chuang et al., 2019). Additionally, nlrp3-mediated caspase-1 typical inflammatory pathway leads to the formation of active IL-1β, IL-18, IL-1α, and HMGB1, while the direct substrate of caspase-11’s atypical inflammatory pathway is IL-1α (Bulek et al., 2020), (Toldo and Abbate, 2018), (Wu et al., 2021).

Collectively, the activation of the multiple cytokine pathways described above can result in sudden and acute increase in the circulating levels of various proinflammatory cytokines, and lead to an overactivation of the inflammatory response. Importantly, the breakdown of mechanisms that tightly regulate inflammatory signaling pathways can be the underlying cause of uncontrolled inflammatory responses. Meanwhile, the binding of different receptors and ligands results in signal cascade amplification, which increases the probability of CS. This suggests that in addition to antiviral therapeutics during the initial phase of the infection, appropriate therapies targeting inflammatory signaling pathways and their downstream components, may be required to dampen the risk of CS due to dysregulation of inflammatory responses.

## 3 CCFIAs protects against inflammation by inhibiting proinflammatory cytokines via regulating signaling pathways

CCF was first recorded in Sheng Nong’s Herbal Classic, and listed as a representative medicine for eliminating dampness by bitter and cool. Additionally, CCF is also a powerful and commonly utilized herb documented in several ancient medical books, such as “Jin-Gui Yao-Lue”, and especially used as the monarch drug in many prescriptions, with functions to clear away heat, resolve dampness, purge fire, and perform detoxification. Clinically, it has also been applied for treating diverse inflammatory-related diseases, such as sepsis, diabetes mellitus and ulcerative colitis (Liu et al., 2021), (Xie et al., 2022), (Ran et al., 2019). Moreover, the studies have shown that CCF controls the development of the disease by regulating signaling pathways and cytokine secretion (Wu et al., 2016). Nevertheless, modern research has demonstrated that the major pharmacodynamic components of CCF that exerts biological activity are isoquinoline alkaloids. Which have anti-inflammatory, antibacterial, antioxidant and hypolipidemic effects (Meng et al., 2018).

**FIGURE 3:**
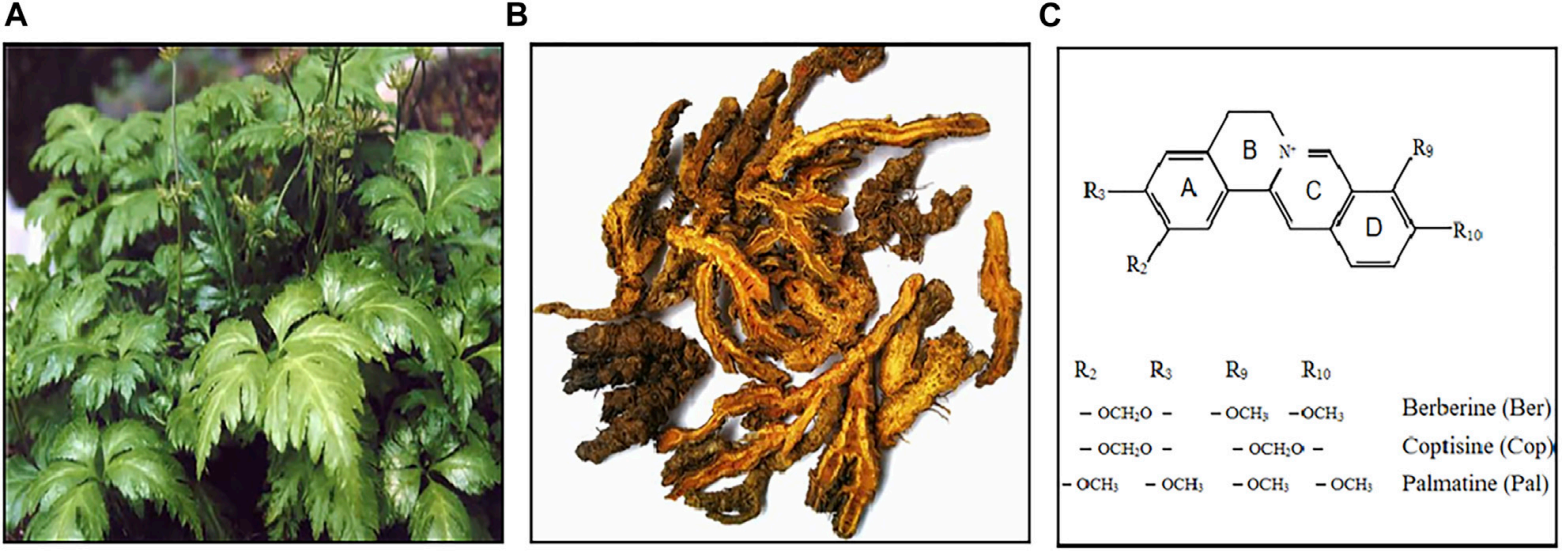
*Coptis Chinensis* Franch. Whole plant **(A)**, dry root **(B)**, chemical structure of berberine, coptisine, and palmatine **(C)**.

Among all isoquinoline alkaloids, BBR, COP and PAL, have the most abundant quantitation, which all belong to protoberberine alkaloids with similar structures, and are the main bioactive components of CCF to exert anti-inflammatory, antibacterial and immunomodulatory effects (Li et al., 2015) (Figure 3). Hence, we propose in (Tables 1, 2) a summarized outcome of available *in vitro* and *in vivo* studies associated with mitigating inflammatory cytokine by CCFIAs, thereby supporting its likely therapeutic benefits against CS. Moreover, these studies demonstrated CCFIAs play a role in inhibiting the production and activation of inflammatory factors by regulating multiple inflammatory signaling pathways (Figure 2).

**TABLE 1:** The reported anti-inflammatory inhibition of Berberine.

Type of study	Model building	Animal or cell culture	Dose	Biologic effects	Molecular mechanism involeves	References
*In vitro*	LPS-treated	RAW264.7	5uM	↓: TNF-α, IL-1β, IL-6	↓p-p65/NF-KB, p-IκBα	Li et al. (2019a)
		RAW264.7	5uM	↓: MCP-1, IL-6, TNF-α	↑Sirt1, ↓p-IKK, p-IκBα	Zhang et al. (2017)
		HDPF	25 μM	↓: IL-1β, IL-6, TNF-α	↓p-p65/NF-KB, p-IKK, p-IκBα	Song et al. (2020)
		MDA-MB-231	25uM	↓: TNF-α, IL-6	↓c-fos, c-jun, p65/NF-KB	Zhao and Zhang et al., 2020
	IL-33-stimulated	RPMCs	10 μM	↓: IL-6, TNF-α, IL-13, MCP-1	↓p-p65/NF-KB, p-IκBα, p-p38	Li et al. (2019b)
	FCA-induced	FLS cells	15–45 µM	↓: TNFα, IL-1β, IL-6, IL-23	↓PI3K/AKT, p-p65/NF-KB, mTOR	Dinesh and Rasool, (2018)
	Primary RA	FLS cells	25uM	↓: TNF-α, IL-6	↓p-p38/MAPK. p-ERK/MAPK	Wang et al. (2019a)
	OX-LDL-treated	THP-1	25uM	↓: IL-6, TNF-α, IL-1β	↑p-AMPK, ↓p-p65/NF-KB	Pei et al. (2019)
	GalN/TNF-α-stimulated	L02 hepatocytes	20 μM	↓: TNF-α, IL-6	↓TLR4/MyD88/NF-κB	Xu et al. (2018)
	IL-4+TNF-α stimulated	BEAS-2B	1 μM	↓: IL-6, CCL11	↓p-STAT6, p-JAK1/2	Ma et al. (2020)
	CD and JAS induced	Rabbit articular chondrocytes	50uM	--	↓p-Akt, p-p38	Yu et al., 2016
	IL-1β-stimulated	Rat chondrocyte	25uM	↓: iNOS, COX2, MMP-3, MMP-13, TNF-α, IL-6	↓p-ERK, p-p38, p-JNK	Li et al. (2019c)
	MSU-stimulated	RAW264.7	25uM	↓: IL-1β, TNF-α	↓NLRP3	Dinesh and Rasool. (2017)
	Primary	MDA-MB-231	40uM	↓: TNF-α, IL-1α, IL-1β, IL-6	↓NLRP3	Yao et al. (2019)
	PMA induced	THP-1	10uM	↓: IL-1β	↓TLR4/MyD88/NF-κB, NLRP3	Huang et al. (2018)
*In vivo*	DSS-induced colitis model	C57BL/6 Mice	10 mg/kg	↓: IL-6, TNF-α, IL-1β	↑AKT1/SOCS1, p-p65/NF-κB	Liu et al. (2018)
		Balb/C mice	20 mg/kg	↓: IL-6, IL-1β, IL-17, TNF-α, IFN-γ	↓TLR4/MyD88/NF-κB	Li et al. (2020a)
		SD rats	40 mg/kg	↓: IL-1, IL-1β, IL-6, IL-12, TNF-α, TGF-β, IFN-γ; ↑: IL-4, IL-10	↓p-STAT3, p-p65/NF-κB	Zhu et al. (2019)
		C57BL/6 Mice	50 mg/kg	↓: TNF-α, IFN-γ, IL-1β, IL-5, IL-22, IL-17A, IL-13, IL-23, CCL2, CCL3, CCL4, CCL17, CCL20, CXCX9, CXCL10, CXCL11	↓p-JAK1/2, p-STAT1/3/4/5/6, p-ERK/MAPK, p-AKT	Li et al. (2020c)
	LPS-induced intestinal injury	SD rats	30 mg/kg	↓: TNF-a, IL-1β, NO	↓TLR4, NF-κB	Zhang et al. (2011)
	LPS-induced acute inflammation	C57BL/6 Mice	5 mg/kg	↓: IL-1β	↓NEK7/NLRP3	Zeng et al. (2021)
	Carrageenan-induced paw edema model	Kunming mice	20 mg/kg	↓: TNF-α, IL-1β, IL-6	↓p-p65/NF-KB, p-IκBα	Li et al. (2019a)
	Silk ligature-induced periodontitis model	SD rats	120 mg/kg	↓: TNF-α, IL-1β; ↑: IL-10	↓p-p38, p-p65/NF-κB	Gu et al. (2021)
	Myosin-induced EAM model	SD rats	200 mg/kg	↓: IL-17, IFN-γ	↓p-STAT1, p-STAT3, p-STAT4	Liu et al. (2016)
	WAS-induced IBS model	SD rats	100 mg/kg	↓: IL-1β, IL-6, IFN-γ, TNF-α; ↑: IL-10, TGF-β	↓p65/NF-κB	Yu et al. (2019)
	CSE-induced COPD model	C57BL/6 mice	50 mg/kg	↓: TNF-α, IL-6, TGF-β	↓TGF-β1/Smads	Wang et al. (2019b)
	IMQ-induced psoriasis-like skin inflammation	BALB/c mice	20uM	↓: IL-18, CXCL1, CXCL16	↓p-JAK1/2, p-Tyk2, p-STAT3	Sun et al. (2019)
	Prechiasmatic cistern injection induced SAH model	SD rats	50 mg/kg	↓: IL-1β, IL-6, TNF-α	↑Sirt1, ↓TLR4/MyD88/NF-κB	Zhang et al. (2020)
	Surgery-induced intestinal adhesion model	SD rats	1.5 mg/ml	↓: IL-6, TNF-α, IL-1β	↓p-TAK1, p-JNK, p-p65/NF-KB	Zhang et al., 2014
	Ova-induced asthma models	SD rats	100 mg/kg	↓: IL-1β, IL-4, IL-5, IL-6, IL-13, IL-17	↓p-p65/NF-KB, p-IκBα	Li et al. (2016)
	Acetic-acid-induced neonatal NEC model	C57BL/6 mice	5 mg/ml	↓: TNF-α, NF-κB, IL-6, CXCL1	↓p-PI3K, p-AKT	Fang et al. (2018)
	Smoke-induced airway inflammation model	BALB/c mice	10 mg/kg	↓: TNF-α, IL-1β, MCP-1	↓p38/MAPK, ERK/MAPK	Xu et al. (2015)
	Intraluminal-suture-method induced tMCAO model	C57BL/6 mice	25 mg/kg	↓: TNF-α, IL-1β, IL-6	↓HMGB1/TLR4/NF-κB	Zhu et al. (2018)
	Bovine type II collagen induced CIA model	SD rats	200 mg/kg	↓: TNF-α, IL-1β, IL-6, IL-17, VEGF	↓p-ERK, p-p38, p-JNK	Wang et al. (2014)
	MCD diet induced NAFLD model	C57BL/6 mice	100 mg/kg	↓: ROS, TNF-α	↓p-p65/NF-KB, NLRP3	Mai et al. (2020)
	2,4-dinitrofluorobenzene induced ACD	SD rats	5 mg/kg	↓: IFN-γ, IL-4	↓p-p38	Li et al. (2018b)
	IFA induced EAMG model	Lewis rats	150 mg/kg	↓: IFN-γ、IL-6, IL-17A; ↑: GM-CSF, IL-10	↓p-JAK1/2/3, p-STAT1/3	Song et al. (2022)
	CDE induced SAP model	C57BL/6 mice	10 mg/kg	↓: TNF-α, IL-1β, IL-6	↓p-p38, p-JNK, NF-κB	Choi et al. (2017)

Note: LPS, lipopolysaccharides; HDPF, human dental pulp fibroblast; RPMCs, rat peritoneal mast cells; FCA, Freund’s complete adjuvant; FLS, fibroblast-like synoviocytes; RA, rheumatoid arthritis; OX-LDL, oxidized low density lipoprotein; CD, cytochalasin D; JAS, jasplakinolide; MSU, monosodium urate; DSS, dextran sulfate sodium; EAM, experimental autoimmune myocarditis; WAS, water avoidance stress; IBS, irritable bowel syndrome; CSE, cigarette smoke extract; COPD, chronic obstructive pulmonary disease; IMQ, imiquimod; SAH, subarachnoid hemorrhage; OVA, ovalbumin; NEC, necrotizing enterocolitis; tMCAO, transient middle cerebral artery occlusion; CIA, collagen-induced arthritis; MCD, methionine-choline deficient; NAFLD, nonalcoholic fatty liver disease; ACD, allergic contact dermatitis; IFA, Freund’s adjuvant; EAMG, experimental autoimmune myasthenia gravis; CDE, choline-deficient ethionine-supplemented; SAP, severe acute pancreatitis.

**TABLE 2:** The reported anti-inflammatory inhibition of coptisine and palmatine.

Alkaloids	Type of study	Model	Dose	Biologic effects	Molecular mechanism involeves	Authors
COP	*In vitro*	DNP-IgE/hsa-stimulated RBL-2H3cells	10 uM	↓: IL-4, TNF-α	↓PI3K/Akt	Fu et al. (2018)
		LPS-stimulated RAW264.7	10 uM	↓: IL-1β, IL-6, IFN-γ	↓NF-kB, MAPK, PI3K/Akt	Wu et al. (2016)
		LPS + ATP stimulated RAW264.7	30 uM	↓: TNF-α, IL-1β, IL-18	↓NLRP3	Chen et al. (2017)
	*In vivo*	DSS-induced mouse colitis	100 mg/kg	↓: TNF-α, IFN-γ, IL-1β, IL-6, IL-17, ↑: IL-10, TGF-β	↓p65/IκBα/NF-κB	Wang et al. (2021)
		Western-type-diet-induced C57BL/6J AS model	150 mg/kg	↓: TNF-α, IL-1β, IL-6	↓NF-κB/p38/JNK	Feng et al. (2017)
		OVA-induced mice Allergic rhinitis model	100 mg/kg	↓: IL-4, TNF-α	↓PI3K/Akt	Fu et al. (2018)
		Surgery-induced rat I/R model	10 mg/kg	↓: IL-1β, IL-6, TNF-a	↓Rho/ROCK	Guo et al. (2013)
		Carrageenan-induced mouse paw edema model	40 mg/kg	↓: IL-6, TNF-α, IL-1β	↓NF-κB, MAPK	Chen et al. (2017)
		HFHC diet induced obesity-related inflammation	46.7 mg/kg	↓: IL-6, TNF-a	↓TLR4	Zou et al., 2015
		LPS-stimulated RAW 264.7 cells	5 uM	↓: HMGB1	—	Kim et al., 2009
PAL	*In vitro*	LPS-stimulated EpH4-Ev	25 uM	↓: IL-6, IL-1β, TNF-α, COX-2	↓ERK1/2, P38, Akt/NF-кB	Ma et al. (2021)
		LPS-stimulated gEECs	20 ug/ml	↓: TNF-α, IL-6, IL-1β, NO, MMP-2, MMP-9	↓TRIF-NF-κB	Yan et al. (2017)
		HP-induced rat CAG model	40 mg/kg	↓: IL-8, MMP-10, CXCL16	↓ADAM17/EGFR	Chen et al. (2020)
	*In vivo*	DSS-induced mouse colitis	100 mg/kg	↓: IL-1β, TNF-α	↓NLRP3	Mai et al. (2019)
		LPS-induced mice sepsis model	5 mg/kg	↓: IL-6, TNF-α	—	Chen et al. (2017)
		IL-1β-induced chondrocytes OA model	100 mg/kg	↓: TNF-α	↓Wnt/β-catenin	Zhou et al., 2016
		Sham-operated (I/R) injury rats	50 mg/kg	↓: COX-2, iNOS	—	Kim et al., 2009

Note: DSS, dextran sulfate sodium; AS, atherosclerosis; OVA, ovalbumin; I/R, ischemia and reperfusion; HFHC, high fat and high cholesterol; EpH4-Ev, mouse mammary epithelial cells; gEECs, goat endometrial epithelial cells; HP, *helicobacter pylori*; CAG, chronic atrophic gastritis; OA, osteoarthritis.

### 3.1 Modulation of BBR on signaling transductions of inflammatory pathways

The mechanism of BBR regulation on pro-inflammatory cytokines has been extensively investigated and engages TLR signaling and three main inflammatory signaling pathways include NF-κB, JAK/STAT, and MAPK (Li et al., 2019), (Wang et al., 2019), (Sun et al., 2019). Additionally, the inhibition for NLRP3 inflammasomes also plays a significant role in its anti-inflammatory effect (Yao et al., 2019). In brief, the mechanism of BBR is mainly through direct inhibition of multiple inflammatory pathways, or via regulation of one signaling pathway to restrain another, or indirectly by modulation of pathway-related genes.

Numerous studies published in recent years have shown that upregulation of the NF-κB signaling pathway is associated with the generation and development of several inflammatory diseases, and it plays a dominant part in the hyperinflammatory response and CS activation (Acar et al., 2018). However, many studies have shown that BBR could directly inhibit the activation of NF-κB signaling pathway by downregulating NF-κB expression (p65/p50 subunits), retarding the phosphorylation and degradation of IκB, and suppressing the NF-κB translocation from cytoplasm to the nucleus (Li et al., 2019), (Yu et al., 2019), (Song et al., 2020), (Li et al., 2016). On the other hand, BBR inhibited the NF-κB pathway by regulating other signaling molecules. For example, BBR suppressed the expression of TLR4 and downregulated MyD88, exerting the broad-spectrum anti-inflammatory actions via downregulation of TLR4-MyD88-NF-κB pathway (Xu et al., 2018), (Zhang et al., 2011), (Li et al., 2020). Moreover, BBR significantly restrained neuroinflammation and brain injury via the HMGB1/TLR4/NF-κB signaling pathway (Zhu et al., 2018). Nevertheless, how BBR modulated HMGB1/NF-κB pathway remains unknown. Importantly, BBR was reported a potent Sirt1 activator, and it could significantly upregulate Sirt1 expression while restrain NF-κB activation (Zhang et al., 2020), (Zhang et al., 2017). In addition, BBR also induced activation of AKT1/SOCS1 and AMPK, thereby inhibiting phosphorylation of NF-κB (Liu et al., 2018), (Pei et al., 2019).

MAPK pathway consists of three well-known serine–threonine protein kinases, including extra-cellular receptor-activated kinase (ERK), p38, and c-Jun N-terminalkinase (JNK) (Seger and Krebs, 1995). The JNK and p38 pathways play a pivatal role in inflammation and tissue homeostasis, more importantly, a study of the effect of p38/MAPK inhibitors on SARS-CoV infected mice reported an 80% increase in survival after treatment (Grimes and Grimes, 2020). It has been reported that BBR inhibited pro-inflammatory responses via directly suppressing the phosphorylation of MAPK, including ERK, p38 and JNK (Wang et al., 2014), (Li et al., 2019). Interestingly, BBR does not always regulate the three kinases simultaneously. In two studies, BBR interfered the phosphorylation of ERK and P38, but not JNK, thereby restraining neutrophilic infiltration and inflammatory cytokine production (Wang et al., 2019), (Xu et al., 2015). Notably, BBR reduced gene expression of microRNA-21, a key regulator of inflammatory cell infiltration and mast cell recruitment, through downregulation of the p38 signaling pathway, ultimately resulting in counteracting allergic inflammation (Li et al., 2018).

JAK/STAT pathway has multiple members, namely four Janus kinases (JAK1-3 and TYK2) and seven STATs (STAT1-4, 5a, 5b, and 6) (Leonard and O'Shea, 1998), (O'Shea et al., 2015). Several studies have reported that BBR inhibited the release of downstream inflammatory cytokines including IFN-γ, IL-6, IL-17A, IL-18, TNF-α, and IL-1β by directly preventing phosphorylation of JAK and STAT (Liu et al., 2016), (Ma et al., 2020), (Song et al., 2022). Importantly, there is growing evidence that JAK-STAT signaling pathway plays a critical role in Th1 and Th17 differentiation and cytokine secretion, and that BBR can suppress TH1/TH17-mediated inflammatory responses by modulating the JAK/STAT signaling pathway. In experimental autoimmune myocarditis (EAM) and experimental autoimmune myasthenia gravis (EAMG) rat models, BBR was found to inhibit autoimmune-induced Th17 and Th1 responses by downregulating STAT and JAK phosphorylation respectively, and eventually led to decreased expression of pro-inflammatory cytokines and chemokines (Liu et al., 2016), (Song et al., 2022). Furthermore, a study provided the new underlying molecular mechanism of BBR’s modulatory action on the JAK/STAT signaling pathway. Li et al. (2020c) announced that BBR prevented the phosphorylation and activation of JAK/STAT members by suppressing Oncostatin M, whose functions as inducing the expression of inflammatory genes, and participates in mucosal inflammation and tissue damage.

Even after its well-established anti-inflammatory potential, the effect of BBR on NLRP3 inflammasome stimulation and downstream pathways has still not elucidated completely. Some studies have shown that BBR significantly ameliorated NLRP3 inflammasome activation and the subsequent pyroptosis process by directly reducing NLRP3, GSDMD-N expression, and caspase-1 activity (Mai et al., 2020), (Dinesh and Rasool, 2017), (Yao et al., 2019). In addition, BBR was also observed to reversed NLRP3 inflammatory assembly by inhibiting the TLR4/Myd88/NF-κB signaling pathway (Huang et al., 2018). Prior to this, few studies have demonstrated that TLR4/Myd88/NF-κB is involved in the regulation of NLRP3 inflammasome. It is worth noting that a study for the first time elucidated the direct binding target of BBR is NIMA-related kinase 7 (NEK7), rather than inhibiting the NF-κB and TLR4 pathways (Zeng et al., 2021). BBR could directly prevent the NEK7-NLRP3 interaction via methylenedioxy binding to the R121, which is residue of NEK7 and located exactly in the key interaction domain of NEK7-NLRP3, therefore the inhibition of BBR for NEK7-NLRP3 is specific, in other words, BBR did not inhibit the activation of IL-1β induced by other inflammasomes including AIM2 or NLRC4 (Zeng et al., 2021), (Sharif et al., 2019).

In addition to regulating these major inflammatory signaling pathways, BBR can also target other signaling molecules. For example, BBR effectively restrained the over-activation of TGF-β1/Smads signaling to attenuate the airway inflammation of chronic obstructive pulmonary disease (Wang et al., 2019). Furthermore, PI3K/Akt is also an important target for inhibiting inflammatory responses (Dinesh and Rasool, 2018), (Fang et al., 2018). Dinesh et al. found that BBR has a therapeutic effect on IL-21/IL-21R mediated signaling pathway in RA pathogenesis by inhibiting the PI3K/Akt signaling pathway and downstream elements (Dinesh and Rasool, 2018). It is worth noting that the regulatory effect of BBR on all the above signaling pathways was not single-targeted, but mostly counteracted excessive inflammatory responses by acting on multiple pathways simultaneously (Gu et al., 2021), (Zhu et al., 2019), (Li et al., 2019).

### 3.2 Modulation of COP on signaling transductions of inflammatory pathways

COP and BBR have the same parent nuclear structure, except that two methoxy groups on ring D of BBR are replaced by a methylenedioxy group, resulting in being inferior to BBR in terms of polarity, and less anti-inflammatory and antibacterial activity than BBR (Li et al., 2015). Similarly, COP also exerts modulatory effects on various inflammatory signals, including NF-κB, MAPK, PI3K/Akt pathways and NLRP3 inflammasome (Wang et al., 2021), (Feng et al., 2017), (Fu et al., 2018), (Wu et al., 2019). However, unlike BBR which downregulated TLR4/MyD88/NF-κB pathway to exert broad-spectrum anti-inflammatory effect, COP had no effect on expressions of TLR-4 and Myd88 as well as LPS binding to TLR-4 in LPS-induced RAW264.7 cells, suggesting that COP may not block downstream pro-inflammatory pathways such as NF-κB, NLRP3 and MAPK through TLR-4 signaling (Xu et al., 2018), (Wu et al., 2019). But it is noteworthy that COP could suppress the activation of the NF-κB pathway by directly inhibiting caspase-1 (Wu et al., 2019). As for NLRP3 inflammasome, BBR could block P2X7R activation and interfere with ATP/P2X7 interactions, whereas COP did not inhibit LPS plus ATP-mediated P2X7R overexpression, thus indicating that COP did not prevent NLRP3 inflammasome activation by inhibiting P2X7R (Wu et al., 2019). In addition, COP also inhibited the expression of downstream inflammatory cytokines TNF-a, IL-1β, and IL-6 by restraining MAPK signaling, and similar with BBR, via blocking phosphorylation of p38 and JNK without suppressing activation of ERK in most inflammatory models (Feng et al., 2017), (Chen et al., 2017), (Choi et al., 2017). Unfortunately, there is no in-depth study on the anti-inflammatory role of COP in regulating JAK/STAT signaling pathway by far.

### 3.3 Modulation of PAL on signaling transductions of inflammatory pathways

PAL also has the parent nuclear structure of proto-berberine, which differs from BBR in that a methylenedioxy on the A ring is replaced by two methylene groups, so they both have similar biological activities of anti-inflammation, antibacteria and immunomodulation (Li et al., 2015). Similar with BBR and COP, PAL also exerted anti-inflammatory effects by inhibiting NF-κB (Yan et al., 2017), (Ma et al., 2021). Of note, Yan et al. found PAL treatment downregulated the gene levels of TLR4, CD14 and TRIF in TLR4 signaling pathways, but it did not affect MyD88 expression. In other words, PAL downregulated NF-κB expression and inhibited NF-κB by restraining TRIF-dependent TLR4 pathways, which was different from the effect of BBR on inhibiting the TLR4-MyD88-NF-κB pathway (Li et al., 2020), (Yan et al., 2017). Interestingly, inhibition of NLRP3 inflammasome activation by PAL is associated with enhanced mitochondrial autophagy, and Mai et al. (2019) found that PAL activated PINK1/Parkin-mediated mitophagy to inhibit the activation of NLRP3 inflammasomes, thus preventing excessive inflammation caused by NLRP3 inflammasome activation. Similarly, BBR also could inactivate the NLRP3 inflammasome via induction of mitophagy in another BNIP3-dependent manner (Liu et al., 2020). Besides, in a *Helicobacter pylori*-induced induced model of chronic atrophic gastritis (CAG), PAL was shown to inhibits the expression of MMP-10 and IL-8 through the ADAM17/EGFR axis and exerts anti-inflammatory (Chen et al., 2020).

As mentioned in the previous paragraphs, CCFIAs are pleiotropic compounds that all demonstrate regulatory effects on NF-κB, MAPK, Akt signaling pathways and NLRP3 inflammasome. In addition, their regulatory objects and mechanisms are also specific, for example, BBR supresses NLRP3 inflammasome by directly acting on NEK7 (Zeng et al., 2021), COP inhibits inflammatory response by inhibiting Rho/ROCK pathway (Guo et al., 2013), and Pal can directly block ADAM17/EGFR signaling (Chen et al., 2020). Therefore, taken together, we anticipate that CCFIAs modulation of inflammatory pathways could exert potential therapeutic benefits against CS and its associated risks.

## 4 BBR ameliorates CS induced by sepsis and viral infections

David et al. proposed a unifying definition of cytokine storm in their paper “Cytokine Storms”, published in the New England Journal of Medicine, the definition is based on the following judging requirements: elevated circulating cytokine levels, acute systemic inflammatory symptoms, and secondary organ dysfunction (Fajgenbaum and June, 2020). This is a major breakthrough in this area, as there has been no consensus on a definition before. Recent clinical data has shown that infection is probably the most common trigger of CS, in other words, bacterial or viral infections induce the production of multiple cytokines, which result in fever, cell death, coagulopathy, and MOF. However, BBR demonstrated promising therapeutic effects in pathological models of CS caused by infection.

Several studies have confirmed that BBR dramatically attenuated tissue damage and death rate in mice challenged with LPS, *Escherichia coli* (*E. coli*), or caecal ligation and puncture (CLP) induced sepsis (Lee et al., 2017), (Pierpaoli et al., 2021), (Li et al., 2015) (Table 3). In LPS-induced acute lung injury (ALI) mice, Chen et al. (2022) found that BBR improved lung permeability while reduced lung injury. Mechanistically, BBR attenuated the expression of NLRP3 via regulating the Phosphorylate-NF-κB, as well as directly inhibited NLRP3 protein and modulated NLRP3 inflammasome pathways. In another study, HMGB1 was found to be a biomarker of BBR for sepsis. 13-ethylberberine (13-EBR) promoted the activation of AMPK and p38/MAPK to inhibit HMGB1, whose excessive accumulation leds to fatal endotoxemia and sepsis. Furthermore, 13-EBR inhibited the activation of NF-κB by activating AMPK, decreased the levels of HMGB1 and iNOS, and alleviated lung and liver injury (Lee et al., 2017). In addition, BBR reduced immune cell infiltration in lung tissue and improved survival rate in septic mice via inhibiting activation of NF-κB and upregulation of several pro-inflammatory transcription factors, like P-STAT3, KIF4 and Myc (Wang et al., 2020).

**TABLE 3:** The ameliorative effect of berberine on sepsis and viral infections.

Experimental model	Role of berberine	Molecular mechanism involeves	Outcome of the study	References
LPS-induced ALI C57BL/6 mice	Anti-inflammatory; Protected against lung injury	Inhibition of p-NF-κB/NLRP3 signaling pathway; Blocking effects of NLRP3	Suppressed IL-1β, IL-18, IL-6, TNF-α; Promoted IL-10	Chen et al. (2022)
LPS-induced endotoxemic BALB/c mice	Anti-inflammatory, Reduced the severity of organ injury	Activation of AMPK-P38/MAPK; Inhibition of p-P65,p-IκBα,	Suppressed HMGB1; Reduced iNOS	Lee et al. (2017)
LPS plus D-galactosamine-induced sepsis C57BL/6 mice	Anti-inflammatory; Protected against ALI; Improved the survival rate of septic mice	Inhibition of p-IKKα/β, p-IκB, P65; Inhibition of p-STAT3, KIF4, Myc	Suppressed IL-1β, IL-6, TNF-α	Wang et al. (2020)
CLP-induced SAE C57BL/6 mice	Anti-inflammatory; Antioxidant; Alleviated sepsis-induced cognitive impairment	Inhibition of HMGB1/RAGE signaling	Reduced expression of TNF-α, IL-1α	Shi et al. (2021)
CS-induced NS C57BL/6 mice	Anti-inflammatory; Increased the survival rates; Reduced the intestinal injurie	Increase the level of miR-132-3p;Inhibition of FOXA1, p-IκBα,P65	Suppressed IL-1β, IL-6, TNF-α	Li et al., 2021
CLP-induced polymicrobial sepsis SD rats	Anti-inflammatory; Attenuated tissue injury	Reduced expression of TLR2, TLR4; Increased expression of TLR9; Inhibition of NF-κB	Reduced expression of TNF-α, IL-6	Li et al. (2015a)
LPS-induced SCM SD rats	Anti-inflammatory; Reduced the myocardial injury	Reduced expression of TLR4; Inhibition of P65	Suppressed IL-1β, TNF-α	Li et al. (2015a)
CLP-induced sepsis Kunming mice	Attenuated neutrophil tissue infiltration and multiorgan dysfunction	Promoted IL-10; Reduced expression of CCR2	Decreased MPO; Increased expression of KC, MCP-1, MIP-1, MIP-2	Wang et al. (2016)
LPS-induced sepsis BALB/c mice	Attenuated neutrophil tissue; Protected against lung injury; Increased the survival rates	Reduced expression of cPLA2,p-cPLA2	Reduced expression of TNF-α	Zhang et al., 2008
A/FM1/1/47 (H1N1) influenza virus-infected C57BL/6 mice	Anti-viral; Anti-inflammatory, Reduced lung injury	Reduced expression of TLR7, MyD88, P65/NF-κB	Suppressed IFN-γ, IL-1β, TNF-α; Promoted IL-4; decreased the ratios of Th1/Th2 and Th17/Treg cells	Zhang et al., 2008
10LD50 influenza virus-infected BALb/c mice	Anti-viral; Anti-inflammatory, Reduced tissue damage	Promoted expression of LC3, BNIP3; inhibited mtROS generation	Reduced expression of NLRP3; Suppressed caspase-1 activation; Suppressed IL-1β; Up-regulated mitophagy	Liu et al. (2020)
Poly I:C-induced RAW 264.7 cells	Anti-apoptosis; Anti-inflammatory	Reduced expression of p-P38, p-ERK 1/2, p-STAT3, p-IkBα	Suppressed NO, PGE2, Fas, GM-CSF, LIF, LIX, RANTES, MIP-2	Kim et al. (2021a)
COVID-19 spike protein-induced SK-N-SH and CCD-841 CoN cells	Anti-inflammatory; Enhanced cell activity	—	Suppressed TNF-α, IL-6	Gu et al. (2021)

Note: ALI, acute lung injury; KLF-4, Krüppel-like factor 4; CLP, caecal ligation and puncture; SAE, sepsis-associated encephalopathy; CS, cecal slurry; NS, neonatal sepsis; SCM, septic cardiomyopathy; CCR2, Chemokine Receptor 2; KC, keratinocyte-derived chemokine; MIP-1, macrophage inflammatory protein-1; MIP-2, macrophage inflammatory protein-2; MCP-1, monocyte chemoattractant protein-1; cPLA_2_, cytosolic phospholipase A_2_; Poly I:C, polyinosinic-polycytidylic acid; PGE2, Prostaglandin E2, Fas: first apoptosis signal receptor; GM-CSF, granulocyte macrophage colony-stimulating factor; LIF, leukemia inhibitory factor; LIX, lipopolysaccharide-induced CXC, chemokine; RANTES, chemokine ligand 5 (Ye et al., 2020).

In addition to alleviating the systemic inflammatory response, BBR also significantly improved DIC and MOF caused by sepsis (Yuan et al., 2021), (Shi et al., 2021), (Chen et al., 2021). A study has indicated that BBR and the derivatives could attenuate coagulation activation, organ dysfunction and further decreased lethality in bacterial sepsis. The mechanism is that BBR blocked the caspase-11 inflammatory pathway by inhibiting the cytoplasmic translocation of LPS via blocking Msr1, a scavenger receptor that mediates endocytosis of LPS (Yuan et al., 2021). In an experimental model of sepsis-associated encephalopathy (SAE), BBR targeted HMGB1/RAGE signaling to suppress the quantity of inflammatory events of cell factors and astrocyte activation in the cerebrum of SAE mice, thereby alleviating cognitive impairment caused by sepsis (Shi et al., 2021) Moreover, septic cardiomyopathy (SCM) is the most common type of sepsis-related organ dysfunction. Chen et al. (2021) found that BBR reduced myocardial injury in sepsis rats, by inhibiting sepsis-induced TLR4/NF-κB signal pathway activation and decreasing the expression levels of TNF-a, IL-1β and other inflammatory factors.

Apart from serving as monotherapy, BBR can be used in combination with other drugs or as an adjuvant for sepsis-treatment. For example, BBR in combination with yohimbine reduced the tissue concentrations of MCP-1, MIP-1α and MIP-2 in the lung, liver and kidney, thus decreasing neutrophil tissue infiltration and multi-organ damage in CLP induced sepsis (Wang et al., 2016). In *E. coli* induced sepsis mice, BBR alone did not reduce bacterial load in mice, but when combined with imipenem, BBR enhanced its antibacterial effect and improved mouse survival rates. Moreover, BBR counteracted the massive pro-inflammatory effect during sepsis, thus making it suitable as an adjunctive treatment for *E. coli*-induced sepsis (Pierpaoli et al., 2021).

Disseminated viral infections can also trigger severe CS, including SARS coronavirus, such as SARS-CoV-2, and other influenza viruses, like H1N1. BBR has been reported to display antiviral and anti-inflammatory effects on a variety of viral infection models. Polyinosinic polycytidylic acid (poly I:C), a synthetic analog of double-stranded RNA (dsRNA), was used to provoke a hyper-inflammatory reaction in macrophages. Kim et al. (2021) found that BBR significantly restrained the phosphorylation of p38/MAPK, ERK1/2, IkB-α, and STAT3, as well as the production of NO, PGE2 and other inflammatory mediators in poly I:C-induced RAW 264.7 cells. In addition, BBR relieved pulmonary inflammation and reduced necrosis and inflammatory cell infiltration induced by H1N1 viral infection in mice, and the mechanism is related to suppressing of TLR7 signaling pathway (Yan et al., 2018). Likewise, BBR reduced lung injury and mortality in 10LD50 influenza virus-infected BALb/C mice (Liu et al., 2020). A recent study revealed the role of BBR to be either an effective treatment for COVID-19 inflammation or a possible component of a treatment, given that it enhanced the viability of SARS-CoV-2 Spike Protein stimulating cells and decreased the cytokines, such as TNF-α and IL-6 (Gu et al., 2021).

Severe cases of sepsis and viral infection are both important triggers for CS. Collectively, in addition to significantly inhibiting the production of cytokines that induced by infection (IFN-λ, TNF-α, IL-1β, IL-6, IL-8, G-CSF, GM-CSF, VEGF, MCP-1, and MIP-1), BBR also exerts antiviral and antibacterial activities. And mounting evidence witnessed by *in vitro* and *in vivo* studies suggested that BBR could interfere systemic inflammatory responses, organ dysfunction/injury and life-threatening conditions caused by hyperinflammatory response.

## 5 Clinical application of BBR in inflammatory diseases

Clinical studies have investigated the benefits of BBR in human health and diseases involving immune disorders and inflammation of various organs (Table 4). In a clinical trial aimed at investigating the anti-inflammatory effects of BBR in children with diarrhea, Chen et al. found that oral administration of BBR hydrochloride (0.2 g/day, for 1 week) significantly reduced the serum levels of pro-inflammatory factors, including TNF-α and IL-6 (Chen et al., 2015). Importantly, another study has shown that BBR (900 mg/day, for 14 days) also could significantly reverse the changes in IL-6, TNF-α and C-reactive protein (CRP) levels in patients with severe COVID-19 with diarrhoea (Zhang et al., 2021). In another double-blind randomized controlled trial, Li et al. (2010) reported that the patients received 300-mg tablets of BBR orally three times daily for 2 weeks reduced the incidence and severity of radiation-induced acute intestinal symptoms (RIAIS), which was presumed to be an inflammatory process involving in cytokines and reactive oxygen metabolites. Furthermore, a randomized, double-blind phase I trial on patients with ulcerative colitis (UC) has been shown that treatment with BBR (900 mg/day, for 3 months) significantly decreased colonic tissue inflammation, and indicated a trend of decreasing plasma levels of pro-inflammatory cytokines such as TNF-α, IL-2, IL-6, IL-8, and IL-4 (Xu et al., 2020). Collectively, the above four studies demonstrated the improvement of BBR in diarrhea and gastrointestinal inflammation.

**TABLE 4:** Clinical application of berberine.

Study population	Study design	Intervention	Findings	Ref
39 patients with severe COVID-19 with diarrhoea	--	900 mg/d (2 weeks)	increased IL-6, TNF-α and CRP levels	Zhang et al. (2021)
Participants with UC	randomized, double-blind, placebo-controlled	900 mg/d (3 months)	decreased in the Geboes score	Xu et al. (2019)
36patients with seminoma or lymphomas	randomized, double-blind, placebo-controlled	900 mg/d (2 weeks)	significantly relieved Syn-dromes such as anorexia, colitis, diarrhea, proctitis	Li et al. (2010)
90 children with diarrhea	randomized	0.2 g/d (1 week)	decreased the serum levels of TNF-α and IL-6	Chen et al., 2015
130 ACS patients undergoing PCI	randomized	300 mg/d (1 month)	MMP-9, ICAM-1, VCAM-1, IL-6 and MCP-1 were significantly reduced	Meng et al. (2012)
100 patients diagnosed with ST-elevated AMI	randomized	900 mg/d (15 days)	CRP, TNF-α and IL-6 were significantly reduced	Qing et al. (2018)
90 patients with NSCLC	randomized, double-blind, placebo-controlled	20 mg/kg (6 weeks)	reduced the incidence of RILI, decreased TGF-β1 and ICAM-1	Liu et al. (2008)
120 patients with AIS	randomized, double-blind	900 mg/d (3 months)	significantly decreased in IL-6 and MIF level, the TPA and the number of unstable carotid atherosclerotic plaques were significantly lowered	Li et al. (2016)
45 ACS patients	randomized, single-blind	300 mg/d (3 months)	downregulated galectin 3, alleviates ox LDL induced macrophage activation	Pei et al. (2019)
184 patients with NAFLD	randomized, parallel controlled, open-label	1.5 g/d (16 weeks)	reduced blood glucose, triglycerides and cholesterol increased liver inflammation	Yan et al. (2015)
patients with RAS for a minimum of 6 months	randomized, placebo-controlled, double-blind	—	decreased pain intensity, promoted ulcer healing, relieved inflammatory	Jiang et al., 2013

Note: CRP, C-reactive protein; AIS, acute cerebral ischemic stroke; MIF, macrophage migration inhibitory factor; TPA, total plaque area; ACS, acute coronary syndrome; PCI, percutaneous coronary intervention; MMP-9, matrix metalloproteinase-9; ICAM-1, intercellular adhesion molecule-1; VCAM-1, vascular cell adhesion molecule-1; MCP-1, monocyte chemoattractant protein-1; AMI, acute myocardial infarction; UC, ulcerative colitis; NAFLD, non-alcoholic fatty liver disease; RAS, recurrent aphthous stomatitis; NSCLC, non-small cell lung cancer; RILI, radiation-induced lung injury; TGF-β1, growth factor-beta-1.

In another clinical study, treatment with BBR (300 mg, t. i.d., for 30 days) in addition to standard significantly decreased MMP-9, ICAM-1, CRP, IL-6, MCP-1 and VCAM-1 in acute coronary syndrome (ACS) patients, and BBR may become an adjunct therapy for ACS patients following undergoing percutaneous coronary intervention since via its anti-inflammatory effect (Meng et al., 2012). Reportedly, BBR (20 mg/kg, for 6 weeks) significantly reduced the incidence of radiation-induced lung injury (RILI) and decreased the levels of ICAM-1 and TGF-β, which are leading to lung injury (Liu et al., 2008). Additionally, in a randomized and single-blind study, BBR (300 mg, t. i.d, for 15 days) significantly reduced serum levels of CRP, TNF-α and IL-6 in patients with acute myocardial infarction compared with control, indicating that BBR provides cardiac protection against cardiac injury (Qing et al., 2018). Interestingly, patients with hypertriglyceridemia are more likely to trigger CS after COVID-19 infection, while BBR reduced blood glucose, triglycerides and cholesterol by directly regulating hepatic lipid metabolism, which might relate to the activation of hepatic AMPK pathway (Yan et al., 2015), (Fajgenbaum and June, 2020).

However, toxicity and safety should be considered in priority when evaluating drug efficacy. It has been reported that the LD50 value of BBR was, respectively, 329 (oral), 9.0386 (i.v) and 57.6103 (i.p) mg/kg, which indicates BBR has relatively wide range of safety, and the common adverse events were mild rash, occasional nausea, vomiting, and fever (Kheir et al., 2010). On the other hand, the dose-effect relationship determines the effectiveness of the drug. Modern pharmacological studies have demonstrated that BBR (50 mg/kg) can be distributed in the heart, spleen, liver, kidney, brain, intestinal tract, muscle and fat and other tissues after oral administration. Except for the intestinal concentration of 4,000 ng/g, other tissues or organ concentration is 200 ng/g. Of note, there was an obvious non-linear relationship between the concentration of BBR in plasma and the oral dose (Chen et al., 2020), and even the highest dosage applied in animal study actually cannot reach the minimum concentration level used in cell experiments dueing to the poor absorption and low availability. Hence, more investigation is needed to improve the bioavailability of BBR and meanwhile reach the balance between toxicological safety and therapeutic efficacy.

In brief, these clinical trials have demonstrated effects of BBR include antioxidation, immune modulation, lipid-metabolism-modulating. And a wide therapeutic window of anti-inflammatory by targeting multiple organs and targets in inflammation response. However, here are still important issues regarding the therapeutic efficacy of the modulatory effects of BBR on pro-inflammatory signaling pathways, therefore, further high-quality clinical trials are needed to determine and validate the modulatory effects of BBR.

## 6 Future perspectives

As mentioned above, CCFIAs exerts potent anti-inflammatory activities through modulatory effect on several signaling pathways, but most reports focused on reducing inflammatory responses and reversing pathogenesis development through classic inflammatory pathways. However, we should also dig into other novel signaling pathways or other aspects of known signaling pathways, such as Wnt, Notch, and MITA, etc., (Sun et al., 2018), (Keewan and Naser, 2020), (Zhou et al., 2019). The targets of these signaling pathways also provide a reference for studying the mechanism of CCFIAs for CS treatment, which has vital clinical implications for the future development of CCFIAs as CS inhibitors. In addition, compared with agents showing only antiviral or anti-inflammatory activity, CCFIAs may exhibit more promising therapeutic potential with respect to the treatment of CS fueled by infection, but the molecular mechanism of CCFIAs in human body has not been fully revealed, thus further clinical studies are in urgent need to support the clinical application of CCFIAs.

To date, there have been many studies on the efficacy and mechanisms of BBR, COP, and PAL in various models of inflammation, whereas comparatively little attention has been devoted to other isoquinoline alkaloids in CCF, such as jatrorrhizine (JH) and epiberberine (EPI). However, JH has been shown to prevent the progression of rheumatoid arthritis (RA) by controling the intensive inflammation. Qiu et al. found that JH inhibited the production of inflammatory mediators such as TNF-α, IL-1β, IL-6, MMP-2, and MMP-3 by blocking the activation of ERK, P38, and NF-κB signaling pathways, suggesting that JH may play a therapeutic role in RA through regulating multiple targets (Qiu et al., 2018). In addition, EPI in the fingerprints possessed positive and relative higher contribution in the anti-inflammatory of CCF, and might be one of the main anti-inflammatory components (Li et al., 2015).

As described previously, CCFIAs are insoluble in water and extensively metabolized *in vivo*, and accordingly their oral absorption and bioavailability are poor, which may restrain biological activity. In recent years, researchers have tried different strategies to improve oral bioavailability of CCFIAs. For example, when BBR was made into nanosuspension, compared with active pharmaceutical ingredients for BBR, the solubility and pharmacological activity of nanopreparation was enhanced (Wang et al., 2015). Besides, specific nanoformulations will also improve permeability and oral bioavailability, such as solid dispersions, nanoemulsions and liposomes (Li et al., 2017), (Xu et al., 2019), (Li et al., 2018). However, strategies to improve oral bioavailability of CCFIAs have only been validated at animal and cellular levels, lacking robust clinical findings, thus more long-term clinical trials are needed.

## 7 Concluding remarks

Although few findings are showing the effectiveness of CCFIAs treatment in COVID-19 disease, they have been proved to prevent excessive inflammation by modulating inflammatory signaling pathways and their downstream components in various *in vitro* and *in vivo* studies. In addition, the aforementioned evidence demonstrated that BBR ameliorates CS induced by sepsis and viral infection, thereby suggesting the beneficial modulatory effects of CCFIAs in reducing CS.
